# Anti-nociceptive and anti-inflammatory effects of the methanolic extract of *Opuntia humifusa* stem

**Published:** 2017

**Authors:** Bhesh Raj Sharma, Chul Min Park, Jong Won Choi, Dong Young Rhyu

**Affiliations:** 1 *Department of Oriental Medicine Resources Mokpo National University, Muan-gun, Jeonnam 58554, Republic of Korea*; 2 *College of Pharmacy, Kyungsung University, * *Busan 48434, Republic of Korea *

**Keywords:** Opuntia humifusa, Anti-nociceptive, Anti-inflammatory, RAW 264.7 cells

## Abstract

**Objective:**

*Opuntia humifusa *(*O. humifusa*) Raf. has been used for the prevention and treatment of rheumatoid arthritis, inflammation, and cancer. Our study was designed to unveil the anti-nociceptive and anti-inflammatory effects of the methanolic extract of *O. humifusa *Raf stem (OHS).

**Materials and Methods:**

The anti-nociceptive effect was measured by hot plate, acetic acid-induced writhing, and tail flick assays in mice and rats. Moreover, the anti-inflammatory effect was measured by vascular permeability and carrageenan and serotonin-induced paw edema tests in rats. Furthermore, anti-inflammatory effect was also measured using macrophage-like LPS-induced RAW 264.7 cells.

**Results:**

OHS extract inhibited acetic acid-induced writhing (p<0.0001), and delayed the reaction time of mice to the hot plate-induced thermal stimulation (p<0.0001) and tail flick tests (p<0.05). OHS extract attenuated the carrageenan and serotonin-induced paw edema in rats (p<0.001). Similarly, OHS extract significantly decreased Evans blue concentration in acetic acid induced vascular permeability test (p<0.0001), revealing its strong anti-inflammatory effect. Finally, among four different fractions of OHS extract, *n*-butanol fraction strongly decreased NO production (p<0.0001) and iNOS expression in LPS-induced RAW 264.7 cells.

**Conclusion:**

Our results suggest that the methanolic extract of *O. humifusa* stem can be used to develop a therapeutic or supportive drug and/or functional food against pain and inflammation related diseases.

## Introduction

Pain and inflammation are the common features of several diseases, which affect millions of people worldwide (Kidd and Urban, 2001 ▶). Chemical stimulants, tissue damages, and autoimmune conditions induce the release of inflammatory mediators, such as bradykinin, prostaglandins (PGs), histamine, tumor necrosis factor (TNF)-α, interleukin (IL)-1β, and many other factors, at the affected site, leading to unceasing activation and sensitization of primary nociceptors, which are involved in the transmission of nociceptive input (Uceyler et al., 2009 ▶). As soon as peripheral pain receptors are activated, nociceptive pain, which guards the body against potential injury, is observed (Stucky et al., 2001 ▶). Therefore, minimizing the inflammation will be an effective means of interfering with the pain sensitization. Macrophages play key roles in important homeostatic functions through production of various cytokines and growth factors against pain and inflammation (Jou et al., 2013 ▶). Hence, lipopolysaccharides (LPS)-induced macrophages in cell culture system, that release several inflammatory mediators, are the popular models for screening anti-inflammatory effects (Sharma et al., 2017 ▶). 

Modern drugs used for the prevention and treatment of pain and inflammation present toxic side effects and secondary effects (Harirforoosh et al., 2013 ▶). Therefore, scientists have attempted to explore the mysterious effect of plant remedies against pain and inflammation. Plants have been important parts of therapy since ancient times, because they contain a wide variety of secondary metabolites with several biological activities (Iriti and Faoro, 2009 ▶). Therefore, despite the availability of synthetic drugs, there is evidence about the therapeutic value of plants (Koehn and Carter, 2005 ▶). It is reported that plants are the sources of 25% prescribed medications (Rates, 2001 ▶). Therefore, recent studies are focused on finding the functional activities of plants. 

*Opuntia humifusa* (*O. humifusa*) Raf. a member of the family cactaceae, is rich in nutrients, such as minerals, oligo elements, betalains, phenolic compounds, polysaccharides, and several vitamins (Sumaya-Martinez et al., 2011 ▶). Compared with other species of Opuntia, *O. humifusa* (OH) contains high concentrations of polyphenols and flavonoids (Yeddes et al., 2014 ▶). It is used as a component of traditional medicine in Indian, American, Mexican, and Korean culture (Hahm et al., 2015 ▶). OH has long been used in Korean Oriental Medicine to treat diabetes, inflammation, and rheumatoid arthritis (Park et al., 2013 ▶). Therefore, the cultivation of OH has increased in Korea (Jun et al., 2013 ▶). We previously isolated five different flavonoids from the methanolic extract of OH (Park et al., 2007 ▶), and in this study, we endeavored to delineate the edifying effect of the methanolic extract of OH on pain and inflammation using *in vitro* and *in vivo* disease models.

## Materials and Methods

**Plant material**

Stems of *O. humifusa* were harvested from Jindo, Sinan-gun, South Korea, in September 2006. The plant specimen was authenticated by Prof. Hui Kim (Department of Oriental Medicine Resources, Mokpo National University), and a voucher specimen was deposited in our laboratory. Fresh stems of *O. humifusa* (6 kg) were chopped and extracted with MeOH (10 L, three times). The extract was concentrated under reduced pressure and the residue (308 g) was suspended in 2L of water and successively extracted three times with 2 L of hexane, EtOAc, and BuOH. 

**Chemicals and reagents**

RAW 264.7 cells (KCLB 40071) were purchased from Korean Cell line Bank (Seoul, Korea), and RPMI 1640 medium and fetal bovine serum (FBS) were purchased from Hyclone (Logan, UT, USA). LPS, thiazolyl blue tetrazolium bromide (MTT), β-actin (A5441), Evans blue, acetic acid, indomethacin, aspirin, codeine, serotonin, and carrageenan were obtained from Sigma-Aldrich (St. Louis, MO, USA). iNOS (SC-650) was purchased from Santa Cruz Biotechnology (Santa Cruz, CA, USA). All chemicals used in the experiments were of analytical grade and obtained from Sigma-Aldrich.

***In vivo*** **assay**

**Animals**

Male Sprague Dawley rats (150-160 g) and albino mice (30-35 g), purchased from the Hyochang Science Inc. (Daegu, Korea), were maintained under standard light (12hr/12hr light/dark) and temperature conditions (22 ± 2 °C). The animals were acclimatized to the laboratory conditions for one week before the commencement of the experiment. The animals were divided into 4 groups (n = 7): Control, vehicle (water)-treated group; *O. humifusa *Raf stem (OHS)100, treated with OHS extract of 100 mg/kg body weight; OHS200, treated with OHS extract of 200 mg/kg body weight; and a positive control group treated with a standard drug. Animals received daily oral OHS extract for one or two weeks. To measure acetic acid-induced vascular permeability, tail flick, and paw edema tests, animals were treated once before the respective induction. The animals were brought to the laboratory 1 hr before the experiments. All experiments were approved by the guidelines of Laboratory Animal Care and Use Committee of Kyungsung University, South Korea. 

**Acetic acid-induced writhing test**

Acetic acid-induced writhing test was used to find the peripheral analgesic activity of the extract (Witkin et al., 1961 ▶). Writhing was induced by the intra-peritoneal injection of 0.7% acetic acid (0.1 ml/10 g) in saline solution. After acetic acid induction, the numbers of muscular contractions in mice were counted over a period of 20 min. The data represent the total number of writhes observed during 20 min, and is expressed as stretching responses. Aspirin (100 mg/kg) was used as a positive control (Vittalrao et al., 2011 ▶).

**Hot plate method**

In this test, mice were individually placed on a hot plate (UGO Basile, Italy) with temperature adjusted to 54 ± 2 °C. The hot plate latency to the first sign of paw licking or jump response to avoid thermal pain was taken as an index of pain threshold. Codeine (10 mg/kg) was used as a positive control (Vittalrao et al., 2011 ▶).

**Tail flick test**

Rats were placed over tail flick unit (UGO Basile, Italy). Rats’ tails were adjusted in the flush mounted window. Then, infrared beam was passed and the time until flicking was measured. Codeine (10 mg/kg) was used as a positive control (Vittalrao et al., 2011 ▶).

**Acetic acid-induced vascular permeability**

The acetic acid-induced vascular permeability test was performed with a little change in an earlier reported method (Hosseinzadeh et al., 2002 ▶). Here, 0.7% acetic acid (0.1 ml/kg) was injected to each animal via intraperitoneal route. Immediately after 30 min of acetic acid injection, 0.2 ml of 4% Evans blue solution was injected into the tail vein of each rat. Rats were then sacrificed after 20 min. Then, 5 ml of saline solution was injected into the abdominal cavity, and then washing was collected into test tubes. Then, the solution was centrifuged for 10 min at 2000 rpm. The amount of Evans blue leakage into the abdominal cavity (vascular permeability) was determined by measuring the absorbance of the supernatant at 580 nm. The amount of dye leaking out from the abdominal cavity was expressed in micrograms. Indomethacin (10 mg/kg) was used as a positive control. 

**Serotonin and Carrageenan-induced edema**

Here, 0.1 ml of 1% carrageenan in 0.9% normal saline was injected into the right hind paw of rats to induce edema. The degree of the paw edema was measured by plethysmometer (UGO Basile, Italy), 1, 2, 3, 4, and 5 hr after carrageenan injection. The positive control used was Ibuprofen (100 mg/kg). For the serotonin-induced edema test, 0.1 ml of serotonin (1 mg/ml) was injected in right sub-plantar region, pedal volume was noted 6, 12, 18, 24 and 30 min after the injection. Indomethacin (10 mg/kg) was used as a positive control. 

***In vitro*** **assay**

**Cell culture and cell viability assay**

RAW 264.7 cells were cultured in RPMI 1640 medium containing 10% fetal bovine serum. Cell viability was determined by MTT assay. RAW 264.7 cells were seeded in a 96-well plate at a density of 210^4^ cells/well. After 4 hr, different concentrations of OHS extract were added to each well and incubated for 24 hr. Then, MTT solution (final concentration, 0.2 mg/ml) was added to each well and incubated for 4 hr at 37^o^C. Finally, formazan crystals were dissolved by adding dimethyl sulfoxide. The optical density was then measured at 540 nm using a spectrophotometer.

**Measurement of NO production**

Briefly, after treatment of RAW 264.7 cells with the sample and LPS (1 µg/ml) for 24 hr in, the culture supernatant was taken and incubated with Griess reagent for 5 min. Then, the absorbance was measured at 540 nm using a spectrophotometer. (Sharma and Rhyu, 2015 ▶). 

**Western blot analysis**

RAW264.7 cell lysate containing 30 µg of protein was separated using 10% sodium dodecyl sulfate polyacrylamide gel electrophoresis. The separated lysate was then transferred to nitrocellulose sheet in a western blot apparatus (Bio-Rad, Hercules, CA, USA). The nitrocellulose sheet was blocked with 5% skim milk for 1 hr and rinsed with Tris-buffered-saline with Tween (1 mol/L Tris, 5 mol/L NaCl, and 0.1% Tween 20). Then, the nitrocellulose sheet was incubated overnight with iNOS primary antibody followed by incubation with secondary antibody (horseradish peroxidase-conjugated IgG 1:2 000) for 1 hr. The expressed protein was finally measured by analyzing the signal captured in nitrocellulose membrane using chemiluminescent substrate (Sharma et al., 2015 ▶).

**Statistical analyses**

GraphPad Prism 6.0 software was used for data analysis. Data are shown as mean ± SEM. Statistical significance was determined by ANOVA (analysis of variance) and Duncan’s multiple range tests. p values of 0.05 or less were considered as statistically significant.

## Results

**Hot plate test**

**Figure 1.**
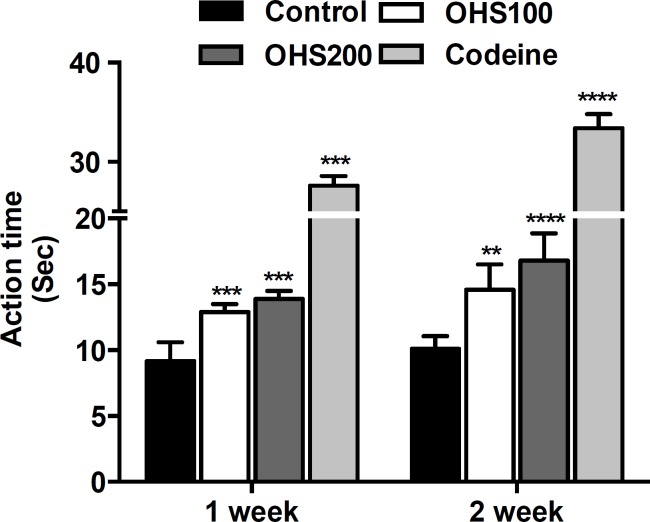
Effect of OHS extract on pain threshold measured by hot plate test. Animals were individually placed on a hot plate with the temperature adjusted to 55 ± 2 °C. The latency to the first sign of paw licking or jump response to avoid thermal pain was taken as an index of pain threshold. Control, vehicle (water)-treated group; OHS100, treated with OHS extract of 100 mg/kg body weight; OHS200, treated with OHS extract of 200 mg/kg body weight. Codeine 10 mg/kg was used as positive control. Data are representative of three independent experiments (mean and SEM). One-way ANOVA. **p<0.01; ***p<0.001; ****p<0.0001

As shown in Figure 1, the hot plate latency for OHS extract was dose and time-dependent. The hot plate latency for OHS100 group mice in one and two weeks was 12.9 and 14.6 sec, whereas the hot plate latency for control group mice was 9.19 and 10.1 sec in one and two weeks, respectively. OHS200 group mice showed significantly increased hot plate latency in two weeks by 66.3% compared to control group mice (p<0.0001). The latency for codeine was 27.6 and 33.4 sec for one and two weeks, respectively.

**Acetic acid-induced writhing response**

**Figure 2:**
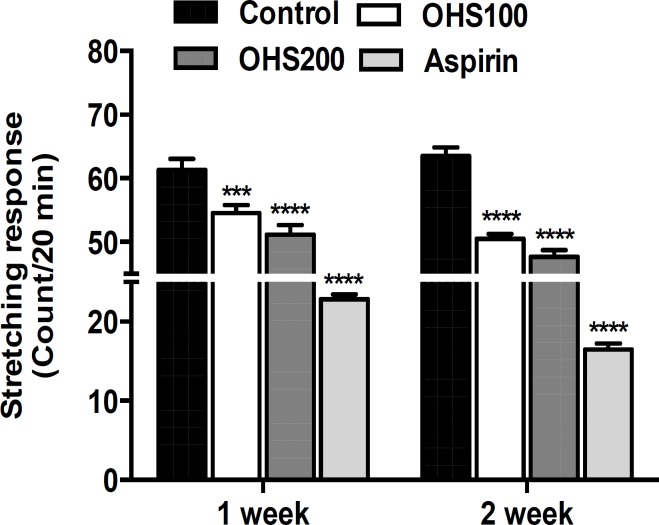
Effect of OHS extract on acetic acid-induced writhing response in mice. The writhes were induced by the intra-peritoneal injection of 0.7% acetic acid (0.1 ml/10 g) in saline solution. The numbers of muscular contractions were counted over a period of 20 min after acetic acid injection. Control, vehicle (water)-treated group; OHS100, treated with OHS extract of 100 mg/kg body weight; OHS200, treated with OHS extract of 200 mg/kg body weight. Aspirin 100 mg/kg was used as positive control. Data are representative of three independent experiments (mean and SEM). One-way ANOVA. ***p<0.001; ****p<0.0001

As shown in Figure 2, OHS extract significantly reduced the number of writhes, which were induced by acetic acid. OHS200 group mice showed 51.2 and 47.7 stretching responses/20 min in one and two weeks, whereas control group mice showed 61.3 and 63.5 stretching responses/20 min in one and two weeks, respectively. OHS200 group mice showed 25% inhibition on the writhing responses compared to control group mice in two weeks (p<0.0001). Positive control mice (aspirin administered) showed 22.8 and 16.5 stretching responses/20 min in one and two weeks. 

**Tail flick test**

**Figure 3:**
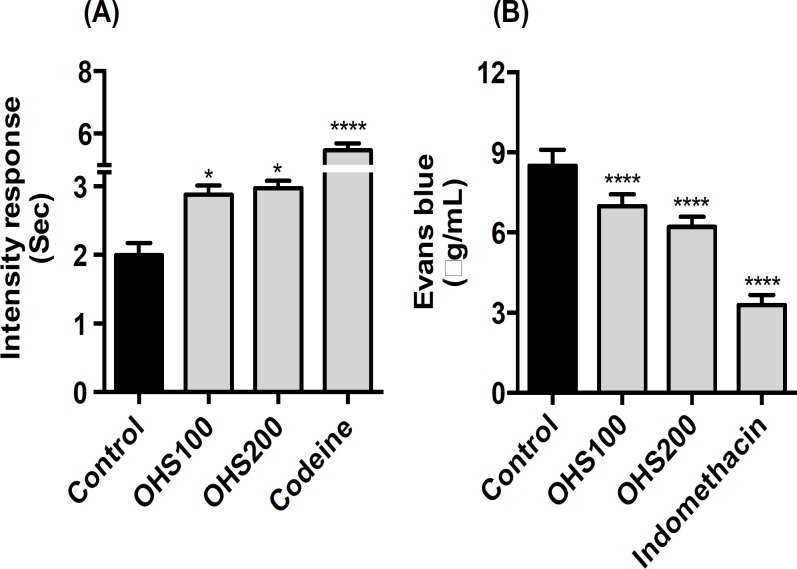
Effect of OHS extract on tail flick test (A) and acetic acid-induced vascular permeability (B). To perform tail flick test, rats were placed over tail flick unit. Rats’ tail was adjusted in the flush mounted window. Then, IRI energy was passed and the time to flicking was measured. For vascular permeability test, the amount of Evans blue leakage in the abdominal cavity was measured as an indicator of inflammation degree. Control, vehicle (water) treated group; OHS100, treated with OHS extract of 100 mg/kg body weight; OHS200, treated with OHS extract of 200 mg/kg body weight. Codeine 10 mg/kg was used as a positive control. Data are representative of three independent experiments (mean and SEM). One-way ANOVA. *p<0.05; ***p<0.001; ****p<0.0001

In tail flick test, OHS extract at only 200 mg/kg body weight significantly increased the intensity response compared to the control group rats. OHS200 produced 25.4 % (reached 2.96 from 2.36) elongation of tail flicking time compared to control group rats. Codeine (positive control) produced elongation of tail flicking time by 131% compared to control group rats (p<0.05) (Figure 3A).

**Vascular permeability test**

The amounts of Evans blue leaked in peritoneum during acetic acid-induced vascular permeability represented the efficacy of the extracts against exudation of fluid from blood vessels. As shown in Figure 3B, Evans blue concentration in control group mice was 8.5 µg/ml. OHS200 group mice showed significantly decreased Evans blue concentration (that is by 26.9%) compared to control group mice (p<0.0001). Indomethacin, the positive control used in the experiment effectively decreased Evans blue concentration to 3.27 µg/ml.

**Table 1:** Effect of OHS extract on serotonin-induced paw edema test

**Group**	**Swelling thickness ( 10** ^-3^ **ml)**				
	**6 min**	**12 min**	**18 min**	**24 min**	**30 min**	
**Control**	6.3±0.05	12.8±0.06	19.3±0.08	26.9±0.05	21.8±0.04	
**OHS100**	6.2±0.04***	12.2±0.08****	18.7±0.05****	25.3±0.03****	20.6±0.04****	
**OHS200**	6.0±0.04****	12.1±0.08****	18.6±0.06****	24.6±0.05****	19.3±0.04****	
**Indomethacin**	5.4±0.04****	10.9±0.04****	13.6±0.04****	15.9±0.03****	12.8±0.03****	

Data are representative of three independent experiments (mean and SEM). One-way ANOVA.

*** p<0.001;

**** p<0.0001. Indomethacin (10 mg/kg body weight) was used as a positive control.

**Table 2:** Effect of OHS extract on carrageenan-induced paw edema test

**Group**	**Swelling thickness ( 10** ^-3^ **ml)**				
	**1 hr**	**2 hr**	**3 hr**	**4 hr**	**5 hr**
**Control**	1.5±0.05	2.7±0.07	3.3±0.06	2.5±0.04	2.0±0.04
**OHS100**	1.4±0.04ns	2.5±0.05****	3.1±0.05****	2.4±0.04**	1.9±0.03*
**OHS200**	1.3±0.04****	2.5±0.04****	2.6±0.05****	2.3±0.03****	1.8±0.03****
**Ibuprofen**	1.0±0.03****	1.2±0.04****	1.6±0.05****	1.2±0.03****	1.0±0.06****

Data are representative of three independent experiments (mean and SEM). One-way ANOVA.

* p<0.06;

** p<0.01;

*** p<0.001;

**** p<0.0001; ns, not statistically significant. Ibuprofen (100 mg/kg body weight) was used as a positive control.

**Serotonin- and carrageenan-induced edema**

The rat hind paw injected with serotonin was used as a model of acute inflammation. As shown in Table 1, OHS100 and OHS200 group rats showed significant inhibition on the hind paw volume from 12 to 30 min. The maximum inhibitory effect of the extract at 200 mg/kg body weight (11.4%) was recorded at 30 min, whereas the standard drug, indomethacin, reduced edema by 41.1% at 30 min. Carrageenan-induced edema was measured up to 5 hr with one-hour interval. As shown in Table 2, OHS100 and OHS200 group rats showed significant inhibition on the pedal volume from 2 to 5 hr. The maximum inhibitory effect of the extract at 200 mg/kg body weight (19.8%) was recorded at 3 hr, whereas the standard drug, ibuprofen reduced pedal volume by 47.3% at 3 hr. 

**NO inhibition and iNOS expression in RAW264.7 cells**

**Figure 4:**
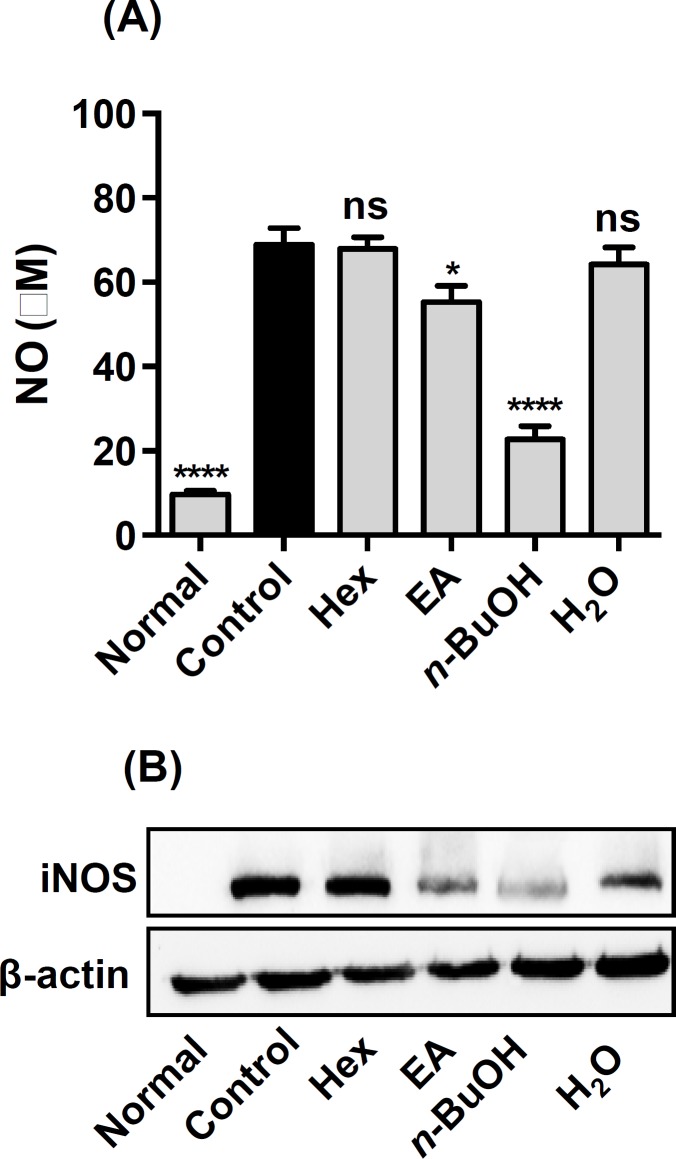
Effect of different fractions of OHS extract on NO production (A) and iNOS expression (B) in RAW264.7 cells. Normal, untreated cells; Control, addition of 1 µg/ml LPS only; Hex, addition of 1 µg/ml LPS plus hexane fraction at 100 µg/ml. EA, addition of 1 µg/ml LPS plus ethylacetate fraction at 100 µg/ml. n-BuOH, addition of 1 µg/ml LPS plus butanol fraction at 100 µg/ml. H2O, addition of 1 µg/ml LPS plus water fraction at 100 µg/ml. EtOAc, butanol, and water fractions were dissolved in water and the hexane fraction was first dissolved in DMSO and then diluted with water. Data are representative of three independent experiments (mean and SEM). One-way ANOVA. *p<0.05; ***p<0.001; ****p<0.0001; ns, not statistically significant

Firstly, we measured cell cytotoxicity of each fraction using MTT assay. No toxicity was observed up to a dose of 100-µg/ml for each fraction (data not shown). As shown in Figure 4, among different fractions of OHS extract, *n*-butanol fraction strongly inhibited NO production (p<0.0001) and iNOS expression in LPS-induced RAW264.7 cells. The *n*-butanol fraction inhibited NO production by 67% compared with only LPS-treated RAW264.7 cells.

## Discussion

Inflammation is associated with many diseases and affects millions of people worldwide (Kidd and Urban, 2001 ▶). Therefore, the prevention and treatment of inflammation will have promising effects in treating those diseases. However, currently available analgesic and anti-inflammatory drugs, such as nonsteroidal anti-inflammatory drugs (NSAIDs) and opiates, are associated with side effects following their long term uses (Harirforoosh et al., 2013 ▶). Herein, we found that the methanolic extract of OHS showed anti-nociceptive and anti-inflammatory effects. Moreover, among different fractions of OHS extract, *n*-butanol fraction strongly decreased NO production and iNOS expression in LPS-induced RAW264.7 cells.

The hot plate test is a suitable method for evaluating the effects of centrally acting, but not peripherally acting, analgesics. The tail flick test is an acute spinally-mediated reflex to noxious thermal stimuli. It is worthy to note that thermal effectiveness of analgesics in the tail flick pain model is highly correlated with the relief of human pain (Kothari et al., 2013 ▶). Numerous folk medicines used for the treatment of inflammation-related diseases, have shown strong stress tolerance capacity in both hot plate and tail flick models of nociception (Vittalrao et al., 2011 ▶). Similar to what had been mentioned in the literatures, in our study, OHS extract prolonged the stress tolerance capacity of the rats in both hot plate and tail flick test, which indicates that the anti-nociceptive activity of the OHS extract possibly resulted from its central and peripheral mechanism. We used two different doses of OHS just to investigate its analgesic and anti-inflammatory effects, and we did not find much difference between these doses in both one and two week treatment. However, in hot plate test, OHS demonstrated stronger effect in two-week group compared to one-week group. These results suggested that the administration of OHS for longer period might prevent the occurrences of nociceptive pain. Codeine, which was used as a positive control, in hot plate and tail flick tests exhibited stronger effect compared to OHS200. 

Intraperitoneal injection of acetic acid stimulates the tissues to produce PG, mainly PGE_2_ and PGF_2α_, and some other endogenous substances, which then excite the pain nerve endings and produce peritoneal inflammation that causes the contraction of the abdominal muscles by extending the rat’s forelimbs and elongating the body (Ricciotti and Fitzgerald, 2011 ▶). The abdominal constriction is related to the sensitization of the nociceptive receptors to endogenous substances (Vinegar et al., 1969 ▶). Our results demonstrated that acetic acid-induced writhing responses are strongly decreased in two-week treated rats compared with untreated control group rats. Herein, we can speculate that the intake of OHS for a longer period might prevent the occurrences of inflammatory consequences in different diseases. Aspirin-treated group (the positive control group) showed strongly decreased writhing response in both one week and two weeks groups. 

Carrageenan, serotonin, and acetic acid-induced animal models are commonly used animal models to evaluate the anti-inflammatory and anti-edemas effects of the natural products (Eddouks et al., 2012 ▶). Edema developed in the paw of the rat after carrageenan injection is a biphasic event (Vinegar et al., 1969 ▶). Histamine and serotonin are released in the initial phase during the first hour of injection, and PG is released in the second phase of edema. Serotonin is one of the important inflammatory mediators and a potent vasodilator. Thus, carrageenan, serotonin, and acetic acid-induced inflammatory models can also stimulate nociceptors (Eddouks et al., 2012 ▶). OHS extract at both doses, significantly decreased swelling at different time points compared to control group rats in both the serotonin- and carrageenan-induced inflammation, in rats. Indomethacin, used as a standard drug in serotonin-induced inflammatory models strongly decreased swelling by 42% at 24 min. Similarly, Ibuprofen, which was used as a positive control, in our carrageenan-induced inflammatory model, decreased swelling by 50% at 5 hr. OHS extract also decreased the Evans blue concentration in acetic acid-induced vascular permeability test. Therefore, we speculate that the anti-inflammatory effects of OHS extract might be related to the inhibition, synthesis, and release of inflammatory mediators. 

Macrophages play an important role in the initiation and amplification of various inflammatory diseases (Jou et al., 2013 ▶). They secrete excessive pro-inflammatory molecules, including NO, which are up-regulated by inducible nitric oxide synthase (iNOS) over-expression during inflammation (Sharma et al., 2017 ▶). Murine RAW264.7 macrophages are popular cellular model to screen the anti-inflammatory effects of natural compounds (Park et al., 2005 ▶). To find effective anti-inflammatory components, we fractionated OHS extract to *n*-hexane, ethyl acetate, *n*-butanol, and water fractions. Only the *n-*butanol fraction decreased NO production in LPS-induced RAW 264.7 cells. Our results agree with previous findings, in which NO inhibitors were isolated from the *n*-butanol fraction of *O. humifusa* fruit extract (Kang et al., 2014 ▶). We isolated five different isorhamnetin flavonoids from the *n-*butanol fraction of OHS extract (Park et al., 2007 ▶). Anti-nociceptive and anti-inflammatory effects of isorhamnetin flavonoid had already been reported (Aquino et al., 2014 ▶). Therefore, we believed that isorhamnetin flavonoids in the methanolic extract of OHS might be responsible for the anti-nociceptive and anti-inflammatory effects of OHS extract. 

In conclusion, our results validate the use of *O. humifusa* in Korean Oriental Medicine against inflammation and rheumatoid arthritis as an analgesic and anti-inflammatory agent. Moreover, our results suggested that the methanolic extract of OHS exhibits anti-nociceptive and anti-inflammatory effects via both the central and peripheral systems. However, isolation of chemical components and their mechanism of action remain to be elucidated.

## References

[B1] Aquino AB, Cavalcante-Silva LH, Matta CB, Epifanio WA, Aqqino PG, Santana AE, Alexandre-Moreira MS, de Araujo-Junior JX (2013). The antinociceptive and anti-inflammatory activities of Aspidosperma tomentosum (Apocynaceae). Sci World J.

[B2] Eddouks M, Chattopadhyay D, Zeggwagh NA (2012). Animal models as tools to investigate anti-diabetic and anti-inflammatory plants. Evid Based Complement Alternat Med.

[B3] Hahm SW, Park J, Oh SY, Lee CW, Park KY, Kim H, Son YS (2015). Anticancer properties of extracts from Opuntia humifusa against human cervical carcinoma cells. J Med Food.

[B4] Harirforoosh S, Asghar W, Jamali F (2013). Adverse effects of non-steroidal anti-inflammatory drugs: an update of gastrointestinal, cardiovascular and renal complications. J Pharm Pharm Sci.

[B5] Hosseinzadeh H, Ramezani M, Fadishei M, Mahmoudi M (2002). Antinociceptive, anti-inflammatory and acute toxicity effect of Zhumeria majdae extracts in mice and rats. Phytomedicine.

[B6] Iriti M, Faoro F (2009). Chemical diversity and defense metabolism: how plants cope with pathogens and ozone pollution. Int J Mol Sci.

[B7] Jou IM, Lin CF, Tsai KJ, Wei SJ (2013). Macrophage-mediated inflammatory disorders. Mediators Inflamm.

[B8] Jun HI, Cha MN, Yang EI, Choi DG, Kim YS (2013). Physicochemical properties and antioxidant activity of Korean cactus (Opuntia humifusa) cladodes. Hort Environ Biotechnol.

[B9] Kang YJ, Kim HY, Lee C, Park SY (2014). Nitric oxide inhibitory constituents from fruits of Opuntia humifusa. Nat Prod Sci.

[B10] Kidd BL, Urban LA (Mechanisms of inflammatory pain). 2001. Br J Anaesth.

[B11] Koehn FE, Carter GT (2005). The evolving role of natural products in drug discovery. Nat Rev Drug Discovery.

[B12] Kothari S, Kushwah A, Kothari D (2013). Involvement of opioid and monoaminergic pain pathways in Aegle marvelos induced analgesia in mice. Indian J Pharmacol.

[B13] Park CM, Kwak BH, Park SH, Hui Kim, Rhyu DY (2013). Comparison of biological activities of Opuntia humifusa and Opuntia ficus indica. Kor J Plant Res.

[B14] Park E, Kum E, Wang C, Park SY, Kim BS, Schuller-Levis G (2005). Anti-inflammatory activity of herbal medicines: inhibition of nitric oxide production and tumor necrosis factor alpha secretion in an activated macrophage-like cell line. Am J Chin Med.

[B15] Park SH, Kim H, Rhyu DY (2007). Flavonoids from the stems of eastern prickly pear Opuntia humifusa, Cactaceae. Appl Biol Chem.

[B16] Rates SM (2001). Plants as source of drugs. Toxicon.

[B17] Ricciotti E, Fitzgerald GA (2011). Prostaglandins and inflammation. Arterioscler Thromb Vasc Biol.

[B18] Sharma BR, Kim HJ, Rhyu DY (2015). Caulerpa lentillifea extract ameliorates insulin resistance and regulates glucose metabolism in C57BL/KsJ-db/db mice via PI3K/AKT signaling pathway in myocytes. J Transl Med.

[B19] Sharma BR, Park CS, Ma SJ, Rhyu DY (2017). Anti-inflammatory effects and mechanisms of Hizikia fusiformis via multicellular signaling pathways in lipopolysaccharide-induced RAW 264.7 cells. Pak J Pharm Sci.

[B20] Sharma BR, Rhyu DY (2015). Lespedeza davuica (Lax) Schindl extract protects against cytokine-induced beta cell damage and streptozotocin-induced diabetes. BioMed Res Int.

[B21] Stucky CL, Gold MS, Zhang X (2001). Mechanisms of pain. Proc Natl Acad Sci.

[B22] Sumaya-Martinez MT, Cruz-Jaime S, Madrigel-Santillan E, Garcia-Paredes JD, Carino-Cortes R, Cruz-Cansino N, Valadez-Vega C, Martinez-Cardenas L, Alanis-Garcia E (2011). Betalin, Acid ascorbic, phenolic contents and antioxidant properties of purple, red, yellow, and while cactus pears. Int J Mol Sci.

[B23] Uceyler N, Schafers M, Sommer C (2009). Mode of action of cytokines on nociceptive neurons. Exp Brain Res.

[B24] Vinegar R, Schreiber W, Hugo R (1969). Biphasic development of carrageenan oedema in rats. J Pharmacol Exp Ther.

[B25] Vittalrao AM, Shanbhang T, Kumari M, Bairy KL, Shenoy S (2011). Evaluation of anti-inflammatory and analgesic activities of alcoholic extract of Kaempferia galangal in rats. Indian J Physiol Pharmacol.

[B26] Witkin LB, Heubner CF, Galdi F, Okeefee E, Spitaletta P, Plummer AJ (1961). Pharmacology of 2-amino-indane hydrochloride (Su-8629): a potent non-narcotic analgesic. J Pharmacol Exp Ther.

[B27] Yeddes N, Cherif JK, Trabelsi Ayadi M (2014). Comparative study of antioxidant power, polyphenols, flavonoids, and betacyanins of peel and pulp of three Tunisian Opuntia forms. Pak J Biol Sci.

